# Transcriptome profile of halofuginone resistant and sensitive strains of *Eimeria tenella*

**DOI:** 10.3389/fmicb.2023.1141952

**Published:** 2023-03-30

**Authors:** Pei Sun, Chaoyue Wang, Yuanyuan Zhang, Xinming Tang, Dandan Hu, Fujie Xie, Zhenkai Hao, Jingxia Suo, Yonglan Yu, Xun Suo, Xianyong Liu

**Affiliations:** ^1^National Key Laboratory of Veterinary Public Health Security, Key Laboratory of Animal Epidemiology and Zoonosis of Ministry of Agriculture, National Animal Protozoa Laboratory and College of Veterinary Medicine, China Agricultural University, Beijing, China; ^2^Department of Pathogen Biology, Guangdong Provincial Key Laboratory of Tropical Disease Research, School of Public Health, Southern Medical University, Guangzhou, Guangdong, China; ^3^Key Laboratory of Animal Genetics, Breeding and Reproduction of the Ministry of Agriculture and Beijing Key Laboratory of Animal Genetic Improvement, China Agricultural University, Beijing, China; ^4^Institute of Animal Science, Chinese Academy of Agricultural Sciences, Beijing, China; ^5^School of Animal Science and Technology, Guangxi University, Nanning, Guangxi, China; ^6^Department of Clinic Veterinary Medicine, College of Veterinary Medicine, China Agricultural University, Beijing, China

**Keywords:** *Eimeria tenella*, halofuginone, transcriptome analysis, DEG, resistance

## Abstract

The antiparasitic drug halofuginone is important for controlling apicomplexan parasites. However, the occurrence of halofuginone resistance is a major obstacle for it to the treatment of apicomplexan parasites. Current studies have identified the molecular marker and drug resistance mechanisms of halofuginone in *Plasmodium falciparum*. In this study, we tried to use transcriptomic data to explore resistance mechanisms of halofuginone in apicomplexan parasites of the genus *Eimeria* (Apicomplexa: Eimeriidae). After halofuginone treatment of *E. tenella* parasites, transcriptome analysis was performed using samples derived from both resistant and sensitive strains. In the sensitive group, DEGs associated with enzymes were significantly downregulated, whereas the DNA damaging process was upregulated after halofuginone treatment, revealing the mechanism of halofuginone-induced parasite death. In addition, 1,325 differentially expressed genes (DEGs) were detected between halofuginone resistant and sensitive strains, and the DEGs related to translation were significantly downregulated after halofuginone induction. Overall, our results provide a gene expression profile for further studies on the mechanism of halofuginone resistance in *E. tenella*.

## Introduction

*Eimeria* is related to malarial parasites and is a major parasitic disease of the intestinal tract of animals and causes huge losses in the poultry industry (Dubey and Jenkins, 2018). Although the current medication against *Eimeria* is quite effective, it has adverse side effects, particularly with regard to the emergence and spread of resistance (Peek and Landman, 2003; Noack et al., 2019). However, there is not yet any unequivocal evidence regarding the drug resistance mechanisms in *Eimeria tenella*. The identification of drug resistance mechanisms is an essential step toward solving drug resistance problems.

Halofuginone, a synthetic derivative of the natural product febrifugine, exhibits potent inhibitory activities against both protozoan parasites and numerous cancer cells (Derbyshire et al., 2012; Zhang et al., 2012; McLaughlin et al., 2014; Herman et al., 2015; Bellini et al., 2020; Cheng et al., 2022). In *Eimeria*, it was shown that halofuginone inhibits the invasion of sporozoites into host cells at an early stage of the life cycle and later disrupts the development of schizonts (Zhang et al., 2012). At present, halofuginone-resistant cases have been reported among different species and have shown that halofuginone resistance is primarily associated with point mutations and copy number variants in the parasite prolyl-tRNA synthetase (PRS) enzyme of *P*. *falciparum* (Keller et al., 2012; Herman et al., 2014, 2015; Hewitt et al., 2017; Jain et al., 2017). In addition, reports on the mode of action have elucidated that halofuginone is an ATP-dependent inhibitor that simultaneously occupies two different substrate-binding sites in prolyl-transfer RNA synthetase (ProRS) (Zhou et al., 2013; Gill and Sharma, 2022; Tye et al., 2022). To date, halofuginone-resistant *Eimeria* strains have been reported, but there is no coherent picture of the mechanism of halofuginone resistance in *Eimeria* (Peek and Landman, 2003; Lan et al., 2017). Resistance hinders the control of coccidiosis in the field; hence, exploring the mechanism of halofuginone resistance would help us to design new anticoccidial drugs.

The development of multi-omics technology provides a bridge to study phenotype-function relationships (Volkman et al., 2012; Hu et al., 2018). To evade different drug pressures, *Eimeria* drug-resistant strains may depend on different mechanisms of gene regulation (Xie et al., 2020). Previous studies have suggested that the differential expression of regulated genes may lead to differences in drug-resistant and drug-sensitive strains, and the current series of studies using transcriptome analysis has been successful in dissecting drug resistance mechanisms (Antony et al., 2016; Ingham et al., 2018, 2020; Xie et al., 2020; Zhang et al., 2022). Here, we performed transcriptome analysis to explore the mechanism of halofuginone resistance in *E*. *tenella*.

## Materials and methods

### Ethics statement

All chickens in this experiment were performed in accordance with the China Agricultural University Institutional Animal Welfare and Animal Experimental Ethical Inspection [Approval number: AW22022202-1-1].

### Animals and parasites

One- to six-week-old Arbor Acres broilers, used for proliferation and halofuginone-resistant line, were purchased from Beijing Arbor Acres Poultry Breeding (Beijing, China). All birds were treated with a coccidia-free diet and water *ad libitum*. The sensitive *E. tenella* Xinjiang strain used was maintained in the laboratory, which is sensitive to the anticoccidial drug halofuginone and used as the parental line. The resistant strains were generated under halofuginone (30 mg/kg) through an experimental evolution strategy and verified through a drug resistance test (Sun et al., 2023). The procedures for collection, sporulation and purification of the parasite were carried out as the previous report (Duan et al., 2020). The cervical dislocation was performed for chickens necessary for sacrifice, which aims to lose consciousness of chickens rapidly.

### Isolation of sporozoites

Sporocysts were extracted from freshly sporulated oocysts after glass-bead grinding. The sporozoites of sensitive strains (HaloS) and halofuginone-resistant strains (HaloR) were purified by the Percoll (Sigma, United States) density gradient method (Dulski and Turner, 1988). To further purify the sporozoites, they were next purified by cellulose DE-52. The viability of sporozoites was tested before *in vitro* culture by trypan blue staining, and sporozoites with >95% viability were used.

### Invasion test of parasites after drug treatment *in vitro*

Madin-Darby bovine kidney (MDBK) cells were used as an infection model. MDBK cells were cultured at 37°C and 5% CO_2_ in 24-well plates with Dulbecco’s modified Eagle’s medium (DMEM, Macgene) supplemented with 10% fetal bovine serum (Macgene, China). For evaluating the efficiency of halofuginone inhibition, approximately 5 × 10^7^ sensitive sporozoites were incubated in DMEM with drug at different concentrations (0, 10, 100 nM, and 1, 5 μM) for 6 h. After the incubation, all samples were washed three times with ice-cold PBS (Solarbio, pH 7.4, Beijing, China). Then fresh MDBK monolayers were seeded into 24-well plates (10^6^/well) and infected with sporozoites (10^5^/well) with different concentrations. All assays were performed in triplicate. At 12 hpi, the cells were washed three times with sterile PBS to wash out uninvaded sporozoites, and new medium was added. Sporozoites were counted at 25 different locations in each well under a microscope. Then the samples were collected and immediately stored at −80°C in TRIzol reagent (Ambion, United States) for the following test using RT-qPCR.

### Comparison of endogenous development *in vivo*

Comparison of the reproductivity of sporozoites treated with halofuginone of different concentrations (0, 10, 100 nM and 1 μM) was tested by measuring oocyst output. For this experiment, 4 groups of 7-day-old chickens were infected with 2 × 10^5^ sporozoites/bird for each strain. Each sample were performed twice.

### RNA-seq

Sporozoites were treated with halofuginone at different concentrations (0, 10, 100 nM and 1 μM) as before and immediately stored at −80°C in TRIzol (Ambion, United States) for the subsequent RNA-seq analysis. Each treatment consisted of three biological replicates. Samples were designated SC, S10, S100, SIU, RC and R1U. The number represents the exposure concentration, and the letters “S” and “R” represent halofuginone-sensitive and halofuginone-resistant strains, respectively. RNA-Seq was performed using an Illumina HiSeq-PE150. The raw reads were subjected to quality control and filtered into clean reads using Trimmomatic-0.38 and then mapped against the *E*. *tenella* reference genome (pEimTen1.1) using STAR aligner (v.2.7.10a). The sorted BAM files were then used for read count *via* featherCounts (v2.0.3). Differentially expressed genes (DEGs) were identified using the R package DESeq2 (Anders and Huber, 2010). Adjusted *p* values were calculated using the Benjamini and Hochberg methods to control the false discovery rate. The standard for screening DEGs was an adjusted *p* < 0.05 and a | Log2 (fold change) | ≥ 1. Functional enrichment analysis was mainly conducted through Gene Ontology (GO) annotation using ClusterProfiler (v 4.0.5).

### qRT–PCR

Total RNA was isolated by using TRIzol (Ambion, United States) according to the manufacturer’s instructions. RNA purity was checked using a NanoPhotometer spectrophotometer (IMPLEN, Los Angeles, CA, United States). cDNA of different samples was also prepared from 100 to 500 ng total RNA using the HiScript III 1st Strand cDNA Synthesis Kit (+gDNA wiper) (Vazyme, Nanjing, China) and random hexamers. Quantitative PCR was performed on Stepone using PerfectStart Green qPCR SuperMix (+Dye I) (TransGen Biotech, Beijing, China). For the evaluation of the efficiency of invasion, transcription was quantified from cDNA (RT-qPCR) using specific primers for the sporozoite (Fw_SP and Pv_SP), together with actin (Marugan-Hernandez et al., 2020). For verification of the RNA-seq data, primers specific for 10 genes were designed for different exons to avoid the amplification of genomic DNA (Supplementary Table 1). The expression of each gene was normalized to the reference gene actin. Relative expression levels were calculated according to the 2^−ΔΔCT^ method.

## Results

### The effects of halofuginone on invasion and endogenous development can be assessed *in vivo* and *in vitro*

**Figure 1:**
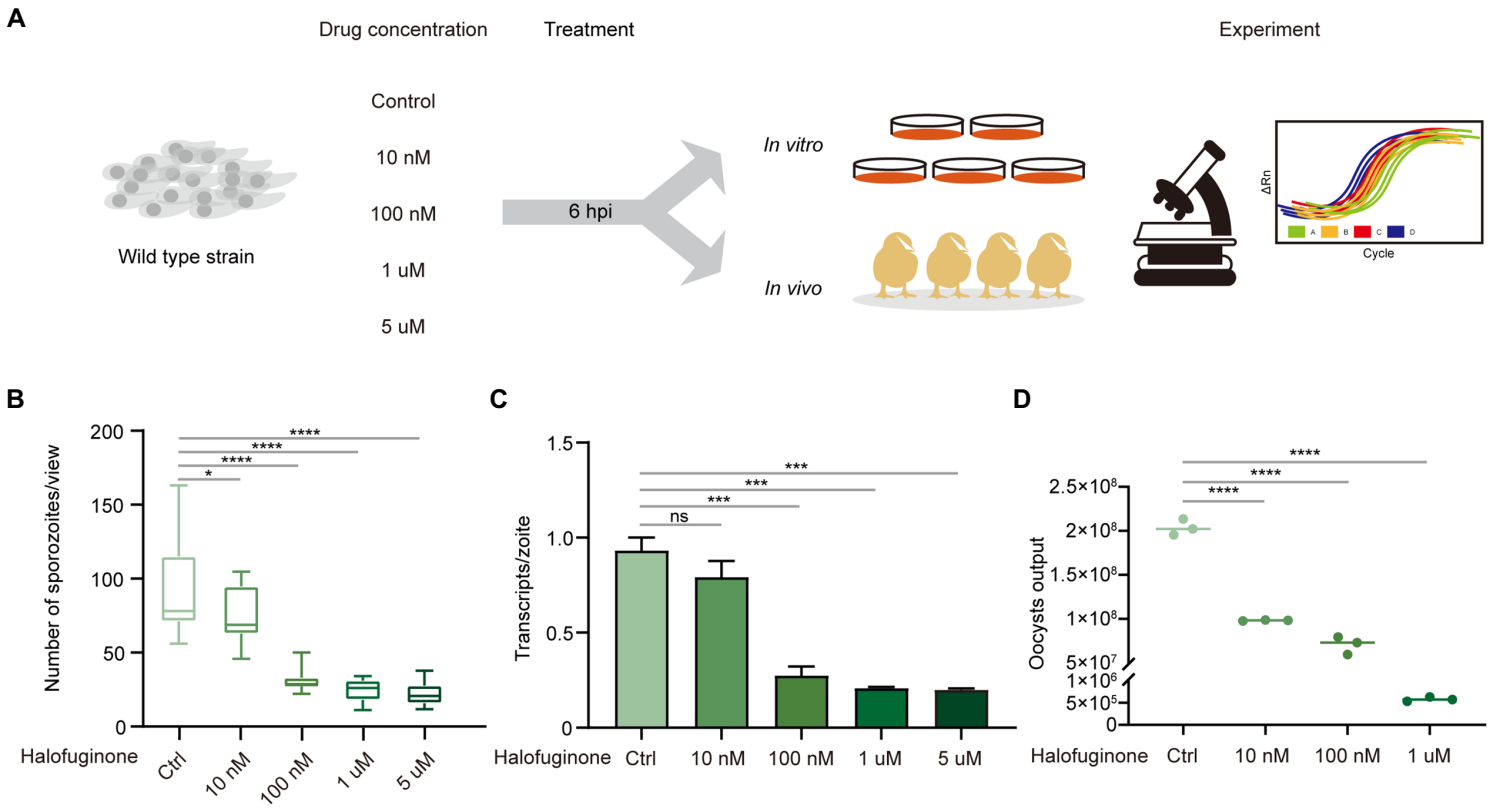
Halofuginone affects invasion and endogenous development in *E. tenella*. **(A)** Schematic illustration of the experimental design. The fresh sporozoites were extracted and treated with different concentrations (0, 10, 100 nM and 1, 5 μM) of halofuginone for 6 h. Designed experiments to detect the difference between different groups *in vivo* and *in vitro*. **(B)** The effect of halofuginone on the invasion efficiency for *E. tenella* in MDBK cells. Sporozoites were counted at 25 different locations in each well under a microscope and each sample was performed with triplicates. ***p < 0.001. **(C)** Detection of changes in invasion by RT-qPCR when sporozoites are preincubated with different concentrations of halofuginone (0, 10, 100 nM and 1, 5 μM). Values represent the mean ± SEM from five independent experiments performed in triplicates. ***p < 0.001. **(D)** Comparison of the number of oocysts output among different concentrations (0, 10, 100 nM and 1 μM). Each sample was performed twice. ***p < 0.001.

To better investigate the effects of halofuginone on the development of *E*. *tenella*, we performed experiments *in vivo* and *in vitro* using different halofuginone concentrations (Figure 1A). Because halofuginone inhibits the invasion of sporozoites, we tested the efficiency of inhibition by counting the number of sporozoites and conducting RT-qPCR analysis *in vitro*. Our data showed that the number of sporozoites gradually decreased with increasing concentration of halofuginone and the number of transcripts per zoite corresponding to sporozoite-specific target SP25 also decreased after drug treatment (Figures 1B,C). To further evaluate whether endogenous development was inhibited, we also compared the number of oocyst outputs among different groups, and the trend was consistent with that *in vitro* (Figure 1D). Overall, halofuginone affected the invasion and endogenous development of *E*. *tenella*.

### Differential expression of genes related to invasion *in vitro* under different concentrations of halofuginone

**Figure 2:**
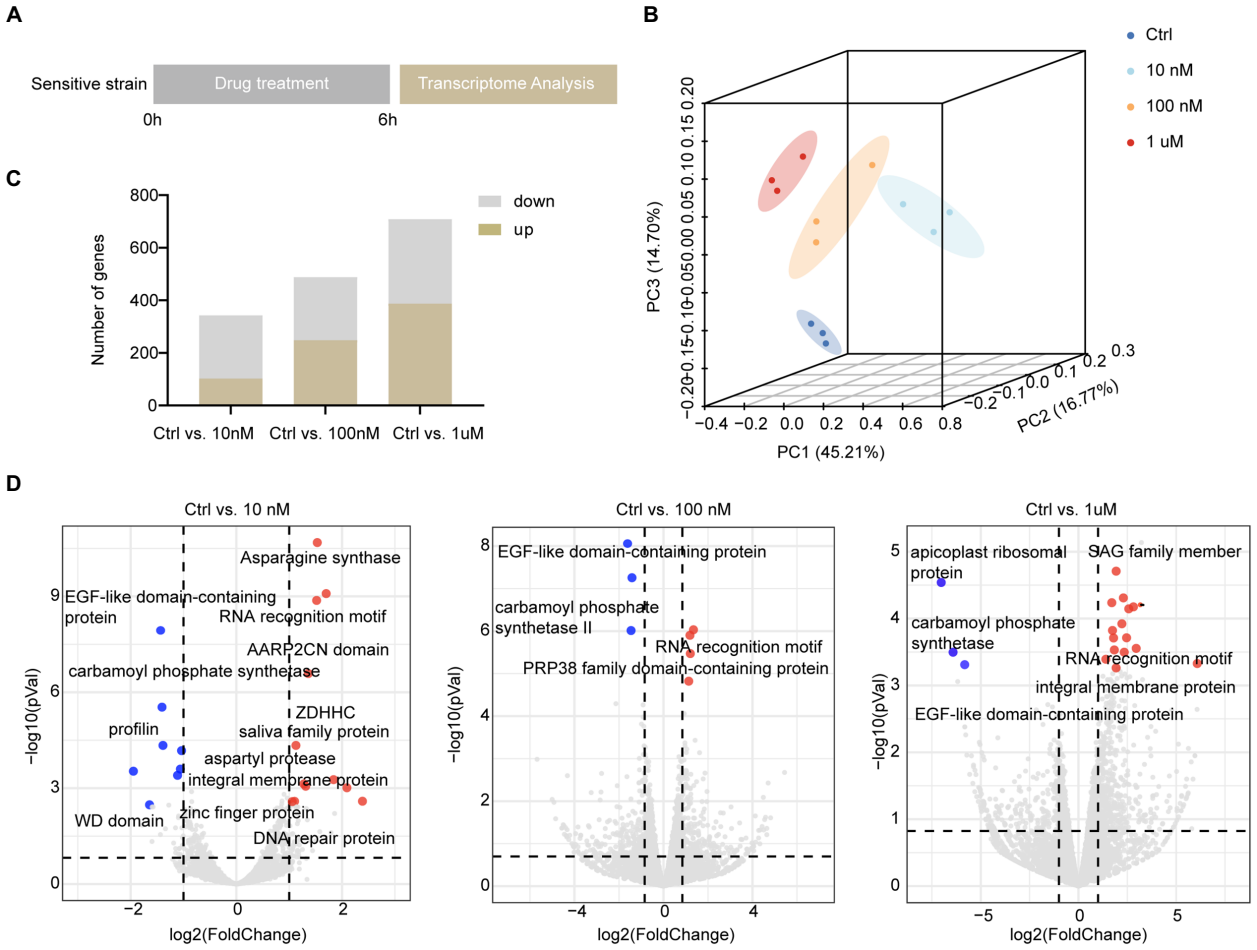
Overall description of transcriptome data among different concentrations in sensitive *E. tenella*. **(A)** Schematic illustration of the halofuginone treatment and sampling strategies. Fresh sporozoites of sensitive *E. tenella* strains were treated with different concentrations (0, 10, 100 nM, and 1 μM) of halofuginone 6 h, respectively. Total RNA was then subjected to RNA-seq. **(B)** PCA plots for transcriptome samples labeled by different halofuginone concentrations. **(C)** Comparison of up-regulated and down-regulated numbers of DEGs under different concentrations, and a gene with a *value of p* less than 0.05 and a two-fold change was considered as a DEG. **(D)** Volcano plots showing the proportion of DEGs in different groups. The red dots represented up-regulated genes and the blue dots represented down-regulated genes, and the gray dots showed no significance.

As halofuginone primarily targets the sporozoite stage of *Eimeria*, we treated the sensitive strain with halofuginone *in vitro*. To screen the gene expression landscape under different halofuginone concentrations, sporozoites were preincubated with different concentrations and subsequently subjected to RNA-seq to track the dynamic change in genes (Figure 2A). The total clean paired-end reads were mapped to the reference genome (pEimTen1.1) (>90% uniquely mapped). Principle component analyses (PCA) of the transcriptomic profiles showed distinct variants among different groups (Figure 2B). The DEGs were counted, and we found that the number of differentially expressed genes increased gradually with increasing drug concentration (Figure 2C). DEG analysis, adjusting for drug concentrations as covariates, identified different numbers of DEGs among the groups (Figure 2C; Supplementary Datasets 1–3). We evaluated the gene expression pattern among different treatment groups. The comparison among the three groups showed considerable changes in gene expression profiles after drug treatment, although some genes had the same expression pattern between different treatment groups (Figure 2D).

**Figure 3:**
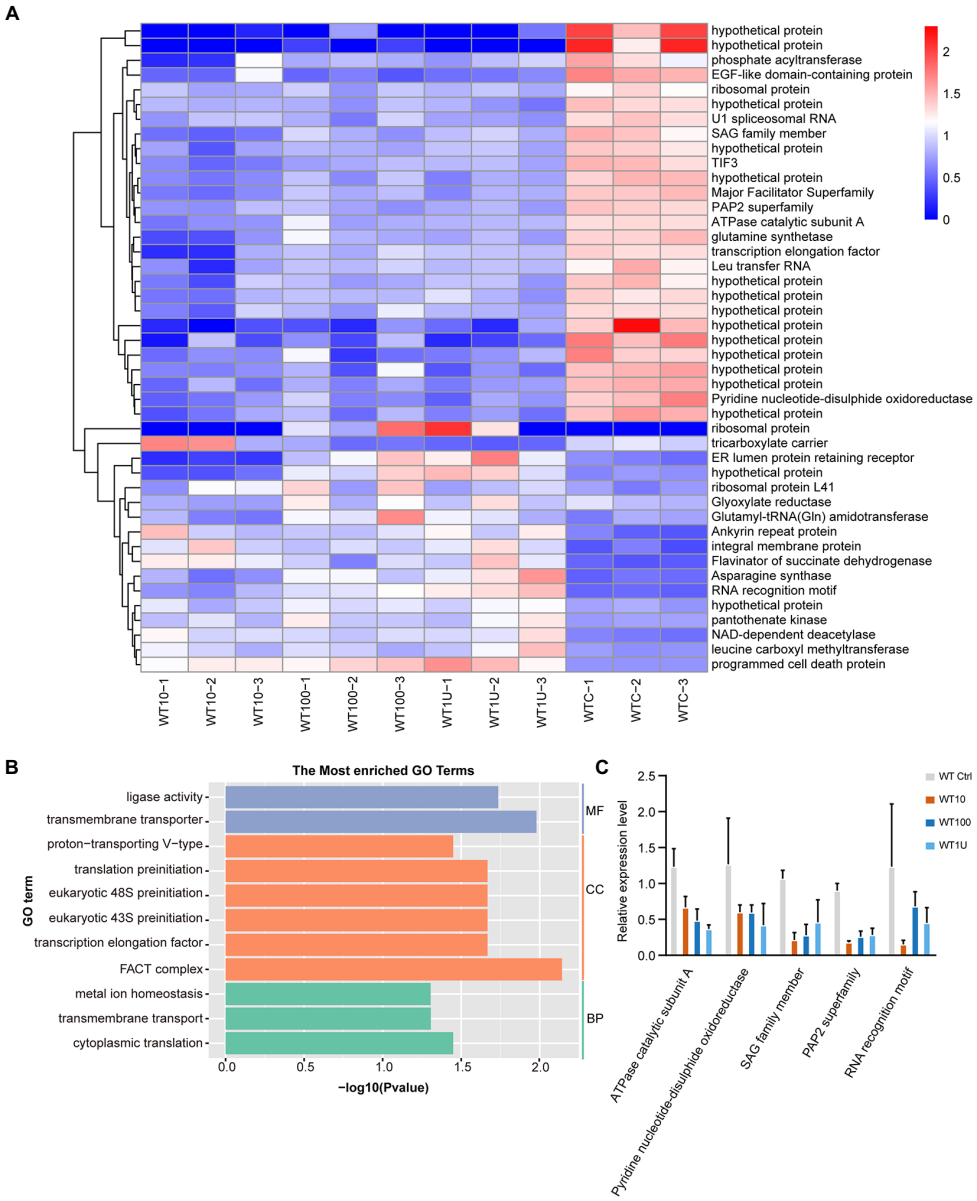
Differential expression of genes shared among sensitive strains after halofuginone treatment **(A)** Heat map of the log_2_ fold change of significantly different genes among three treatment groups. Each treatment consisted of three biological replicates **(B)** Functional enrichment of shared DEGs among sensitive groups with different concentrations of halofuginone. The 11 most extensive GO terms of the three GO categories “molecular function” (blue), “cellular component” (orange), and “biological process” (green) are shown. **(C)** Verification of gene expression by real-time quantitative PCR. The black bars with standard errors indicate fold changes based on the relative expression level determined by qPCR using the 2^−ΔΔCT^ method for three biological replicates.

Hierarchical clustering analysis of the differentially expressed transcripts allowed the identification of groups of co-expressed transcripts for each group. Interestingly, only 44 DEGs were shared among the different sensitive groups (Figure 3A; Supplementary Dataset 4). We hypothesized that the expression of these co-expressed genes was related to halofuginone damage in *E*. *tenella*.

With the exception of 16 genes that showed no significant pattern after halofuginone treatment, 27 genes were downregulated, and one gene related to programmed cell death was upregulated (Figure 3A). As a result of drug pressure, the parasite expression levels of some enzymes, such as those involved in cytoplasmic translation (GO:0002181) and ligase activity (GO:0016874), were significantly reduced (Figure 3B). Besides these enzymes, the expression of transmembrane transporter (GO:0055085, GO:0022857) also downregulates. Five genes were randomly selected to validate the transcriptome data by RT–qPCR analysis and the downregulated genes selected from the transcriptome had the same pattern in the RT–qPCR analysis (Figure 3C). Based on our data, the genes involved in important biological processes were repressed and programmed cell death was significantly activated, which led to parasite death after halofuginone treatment.

### Gene expression changes after halofuginone resistance induction

**Figure 4:**
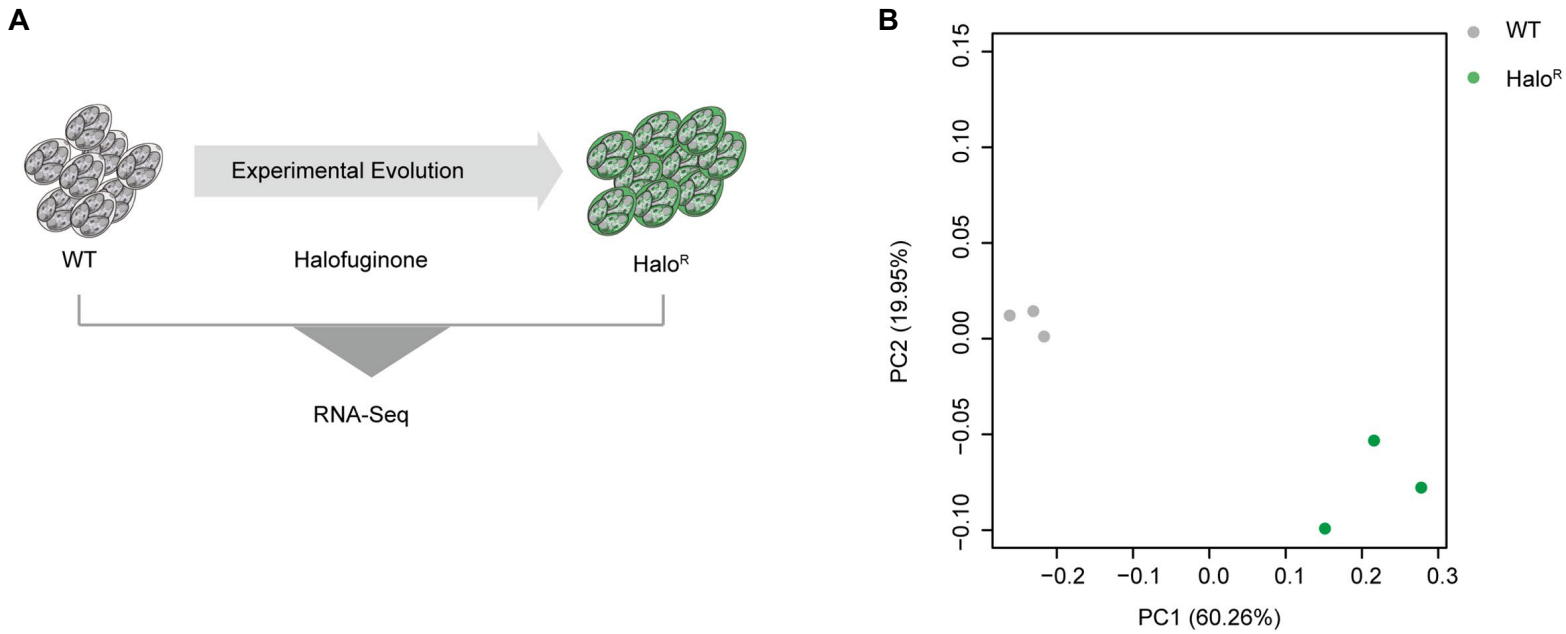
Overview of transcriptome analysis between halofuginone-sensitive strain and resistant *E. tenella* strains. **(A)** Schematic illustration of the induction of halofuginone-resistant strain. **(B)** PCA plots for transcriptome samples labeled by different strains. Gray dots represent wild-type strains while green dots represent halofuginone-resistant strains.

To identify novel mechanisms of resistance mediated at the level of transcription, we subjected halofuginone-resistant and halofuginone-sensitive strains of *E*. *tenella* to comparative transcriptomics using RNA sequencing (Figure 4A). We hypothesized that after obtaining a stable resistance phenotype, only a few gene expression levels change after drug treatment within a short time. Then we compared the transcriptomic data among halofuginone-resistant strains with different concentrations, and there were only 12 DEGs existed (Supplementary Dataset 6). We reasoned that the differences in gene expression among resistant and sensitive strains may drive mechanisms of resistance. PCA was performed to cluster the 6 samples based on gene expression level, and PC1 showed a distinct distance between halofuginone-resistant and -sensitive strains (Figure 4B). Based on our data, there were 1,325 DEGs between the resistant and sensitive groups (Supplementary Dataset 5). Thus, halofuginone had little effect on the gene expression pattern of the drug-resistant group.

**Figure 5:**
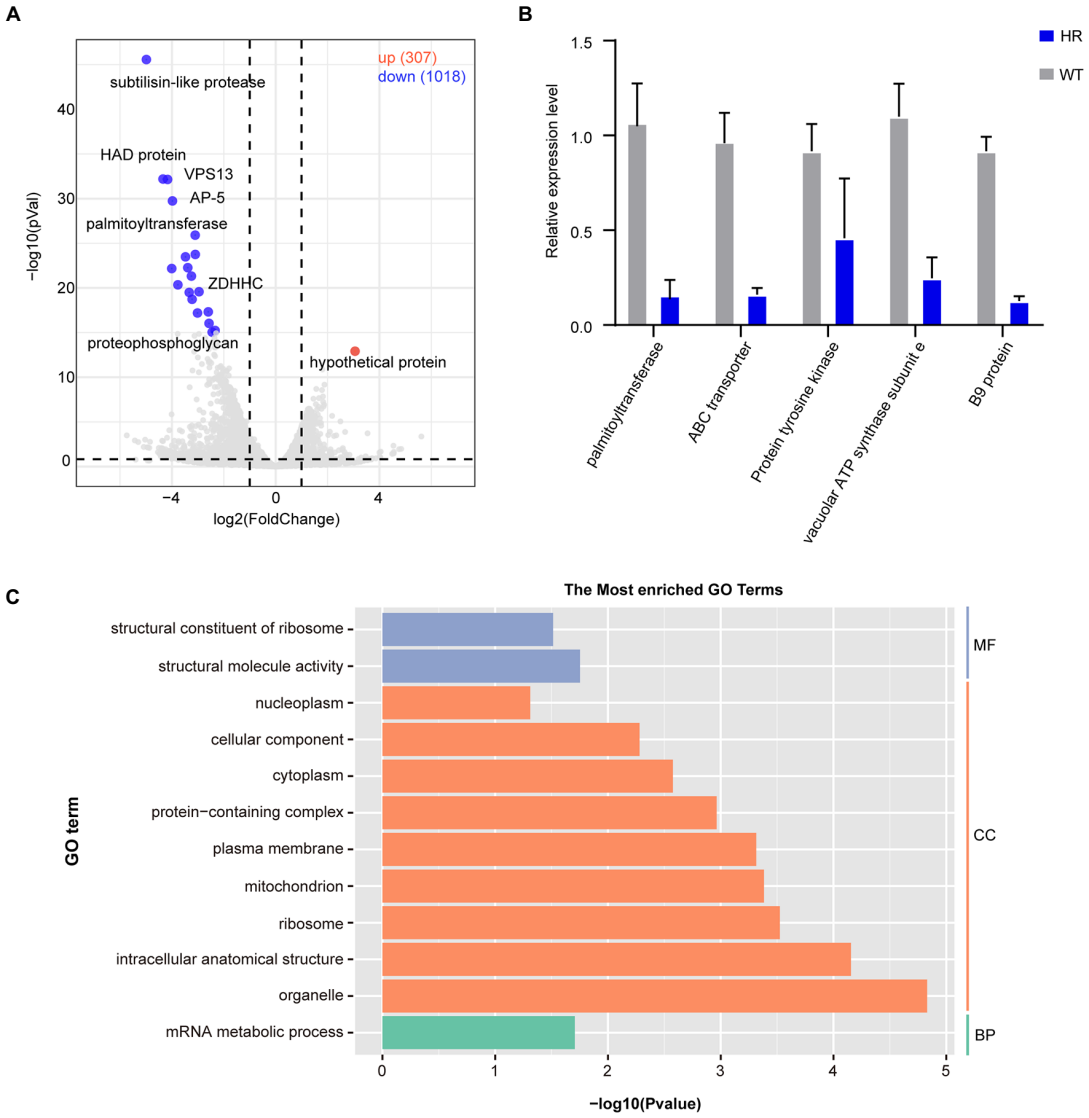
Differential gene expression after halofuginone induction. **(A)** Volcano plot showing the proportion of DEGs between the sensitive group and resistant group. The red dots represented up-regulated genes, the blue dots represented down-regulated genes, and the gray dots showed no significance. **(B)** Verification of gene expression by real-time quantitative PCR. The black bars with standard errors indicate fold changes based on the relative expression level determined by qPCR using the 2^−ΔΔCT^ method for three biological replicates. **(C)** Functional enrichment of DEGs between halofuginone-sensitive and -resistant strains. The 12 most extensive GO terms of the three GO categories “molecular function” (blue), “cellular component” (orange), and “biological process” (green) are shown.

To further explore the expression dynamics of DEGs, a volcano plot was generated, and the results showed that there were a total of 1,325 DEGs of which 1,018 DEGs were downregulated and 307 DEGs were upregulated (Figure 5A). RT–qPCR was performed to verify the transcriptome data (Figure 5B). To further understand the functions of DEGs after halofuginone induction, we performed a GO analysis (Figure 5C). Interestingly, among these downregulated genes, translation-related genes (GO:0016071) and structural constituents of ribosomes (GO:0005840, GO:0003735) were statistically significant, suggesting the downregulation of protein translation in resistant strains (Figure 5C). We also found that the expressions of some genes involved in ATP synthase were decreased (Supplementary Figure 1). These processes are all crucial for parasite protein translation, indicating that protein synthesis was inactive in the halofuginone-resistant strain.

## Discussion

Halofuginone, a traditional Chinese medicine, is used as an anticoccidial drug to control coccidiosis (Zhang et al., 2012). However, the therapeutic utility of halofuginone and its analogs have been stymied by resistance, and the previously unknown mode of action in the parasite has impeded the rational development of drugs with improved pharmacological properties (Peek and Landman, 2003). Our recent work has identified that *EtcPRS*^Mut^ also confers halofuginone resistance in *E. tenella* (Sun et al., 2023). Here, we performed transcriptome analysis to explore the potential halofuginone resistance mechanism in *E. tenella*. As outlined above, we found that the expressions of genes involved in translation and ribosomal proteins were significantly decreased in resistant strain, which represents the potential resistance mechanism of halofuginone in *E. tenella*. In addition, hundreds of DEGs were detected after halofuginone treatment in sensitive groups, and the number of DEGs increased gradually with increasing the concentration of halofuginone. However, only 12 DEGs existed in the resistant group, which indicates that halofuginone has a more significant impact on sensitive strains. Furthermore, we provided a gene expression profile to explore the difference between halofuginone-sensitive and resistant strains, which could help us to further explore the mechanism of anticoccidial drugs in *E. tenella*.

Up to date, one of the great challenges is to identify the functional role of loci that are identified as genomically diverse or under selection in the parasite genome. To explore the further information between phenotype and genotype, additional insight can be obtained from transcriptome analysis (Richardson and Kaye, 2005; Volkman et al., 2012). To explain the mechanism of halofuginone inhibition, we performed transcriptome analysis to screen the gene profile under halofuginone treatment. By screening the transcriptome data in our study, we found that the expression of ATPase catalytic, SAG family member and several other enzymes (glutamine synthetase and pyridine nucleotide-disulfide oxidoreductase) involved in important biological processes were downregulated, and the expression of programmed cell death protein was upregulated. As we all know, enzymes participate in multiple metabolic processes; for example, detailed studies of glutamine synthetase (GS) have shown that it is an ATP-dependent enzyme found in most species, which synthesizes glutamine from glutamate and ammonia (Tardito et al., 2015; Ben Haim et al., 2021). Downregulation of GS may delay cell cycle progression and morphological alteration. In *Leishmania*, GS can be exploited as a potential drug target (Cruzat et al., 2018). In *Leishmania*, it can be exploited as a potential drug target (Kumar et al., 2021). Based on our data, the expression level of programmed cell death protein is upregulated, the result is consistent with our previous work (Figure 1). Previous work reported that autophagy was a potential mechanism for drug-induced parasite killing in *Toxoplasma* and *Eimeria*, and numerous *in vitro* studies have found that drug treatment-induced programmed cell death in trypanosomes and *Plasmodium* spp. (Lavine and Arrizabalaga, 2012; Proto et al., 2013; Charvat and Arrizabalaga, 2016; Zhang et al., 2022). Thus, many successful inhibitors to date have been designed to induce programmed cell death (Kepp et al., 2011). Our comprehensive results indicate that halofuginone damages biological pathways and induces autophagy in *E. tenella*.

Previous studies also found that differential expression of regulated genes may lead to differences in drug-resistant and drug-sensitive strains (Antony et al., 2016; Ingham et al., 2020; Xie et al., 2020; Laing et al., 2022; Okombo et al., 2022; Zhang et al., 2022). Hence, it was expected that long-term selection would lead to transcriptional changes in different genes in the resistant strain. Although there is no evidence regarding the resistance mechanism in *E. tenella*, several mechanisms of drug resistance exist in *P. falciparum* and *T. gondii* (Ariey et al., 2014; Herman et al., 2015; Amato et al., 2017; Palencia et al., 2017; Mishra et al., 2019; Rosenberg et al., 2019; Bellini et al., 2020; Rocamora et al., 2021). As discussed in the previous review, resistance development to toxic drugs in different species shows both important similarities and differences (Hughes and Andersson, 2015). With regard to similarities, it is striking that the mechanisms of resistance are often genetically and functionally similar between these different organisms and cells. In the reports on *P. falciparum* and *T. gondii*, the mechanism of resistance could be summarized as 1) reducing the interaction between the drug and target by mutation, modification, or protection; 2) restricting the internal concentration of the drug by altering efflux or influx; and 3) reducing the concentration of the active drug by altering enzymatic activity (Hughes and Andersson, 2015). Comparing the expression landscape of the halofuginone-resistant and halofuginone-sensitive strains, 1,325 DEGs were identified. In our study, we found that genes involved in the mRNA metabolic process and ribosome proteins were downregulated in the halofuginone-resistant strain. Parasites need a powerful translation system to synthetic protein to complete the complex life cycle (Bennink and Pradel, 2019). In contrast, we found a significant reduction in transcription levels of translation-related genes in resistant strains, which also explains the poor fitness of resistant strains in the fields (Zhang et al., 2022). As previously described, one mechanism by which cells cope with environmental stressors is to downregulate the rate of protein synthesis and increase the selective expression of certain genes for stress adaptation (Han et al., 2020; Kusnadi et al., 2020). In accordance with previous findings, halofuginone acts as a prolyl-tRNA synthetase inhibitor, which ultimately impedes protein translation (Keller et al., 2012; Zhou et al., 2013; Jain et al., 2017). Furthermore, downregulating the process of translation may lead to resistance to halofuginone in *E. tenella*. We hypothesized that mutations that change gene expression can contribute to halofuginone resistance after drug induction. In other species, mutations in *cis*- or *trans-*regulatory elements in the promoter regions of genes of interest and/or changes in the expression level of transcription factors binding to these *cis*- or *trans-*regulatory elements may contribute to drug resistance (Liu et al., 2015; Hu et al., 2021). Unfortunately, detailed information about *cis*- or *trans-* regulatory elements in apicomplexan parasites is limited. Two conserved sequences (RPA: 5’CGGCTTATATTCG, RPB: 5’YGCATGCR) were found in *T. gondii* (Mullapudi et al., 2009). From the DEGs, we only found 7 genes related to translation may be regulated by RPB in *E. tenella* (Supplementary Figure 2). This is interesting for the further study.

In summary, this study highlights the remarkable transcriptome versatility displayed by *E. tenella* for both drug inhibition and resistance mechanisms. This may provide an effective approach to exploring drug resistance mechanisms in *Eimeria*.

## Data availability statement

The data presented in the study are deposited in the NCBI repository (https://www.ncbi.nlm.nih.gov/), accession number PRJNA921718.

## Ethics statement

The animal study was reviewed and approved by China Agricultural University Institutional Animal Welfare and Animal Experimental Ethical Inspection.

## Author contributions

PS: performed experiment, wrote the original draft, and data analysis. CW, XT, and DH: conceptualized project. YZ: data analysis. FX and ZH: generated some strains and cell lines. JS: helped with some general experiments. YY, XS, and XL: funding acquisition and supervision. All authors read and approved the final manuscript.

## Funding

This study was supported by the National Natural Science Foundation of China (31873007 and 32072884) and the National Key Research and Development Program of China (2018YFD0500300 and 2016YFD0501300). XL was supported by the 2115 Talent Development Program of China Agricultural University.

## Conflict of interest

The authors declare that the research was conducted in the absence of any commercial or financial relationships that could be construed as a potential conflict of interest.

## Publisher’s note

All claims expressed in this article are solely those of the authors and do not necessarily represent those of their affiliated organizations, or those of the publisher, the editors and the reviewers. Any product that may be evaluated in this article, or claim that may be made by its manufacturer, is not guaranteed or endorsed by the publisher.

## Supplementary material

The Supplementary material for this article can be found online at: https://www.frontiersin.org/articles/10.3389/fmicb.2023.1141952/full#supplementary-material
