# Supplementary figures and images for: Transcriptome profile of halofuginone resistant and sensitive strains of Eimeria tenella
Source: Front Microbiol. 2023 Mar 30;14:1141952. doi: 10.3389/fmicb.2023.1141952 (PMC10098198; doi:10.3389/fmicb.2023.1141952)

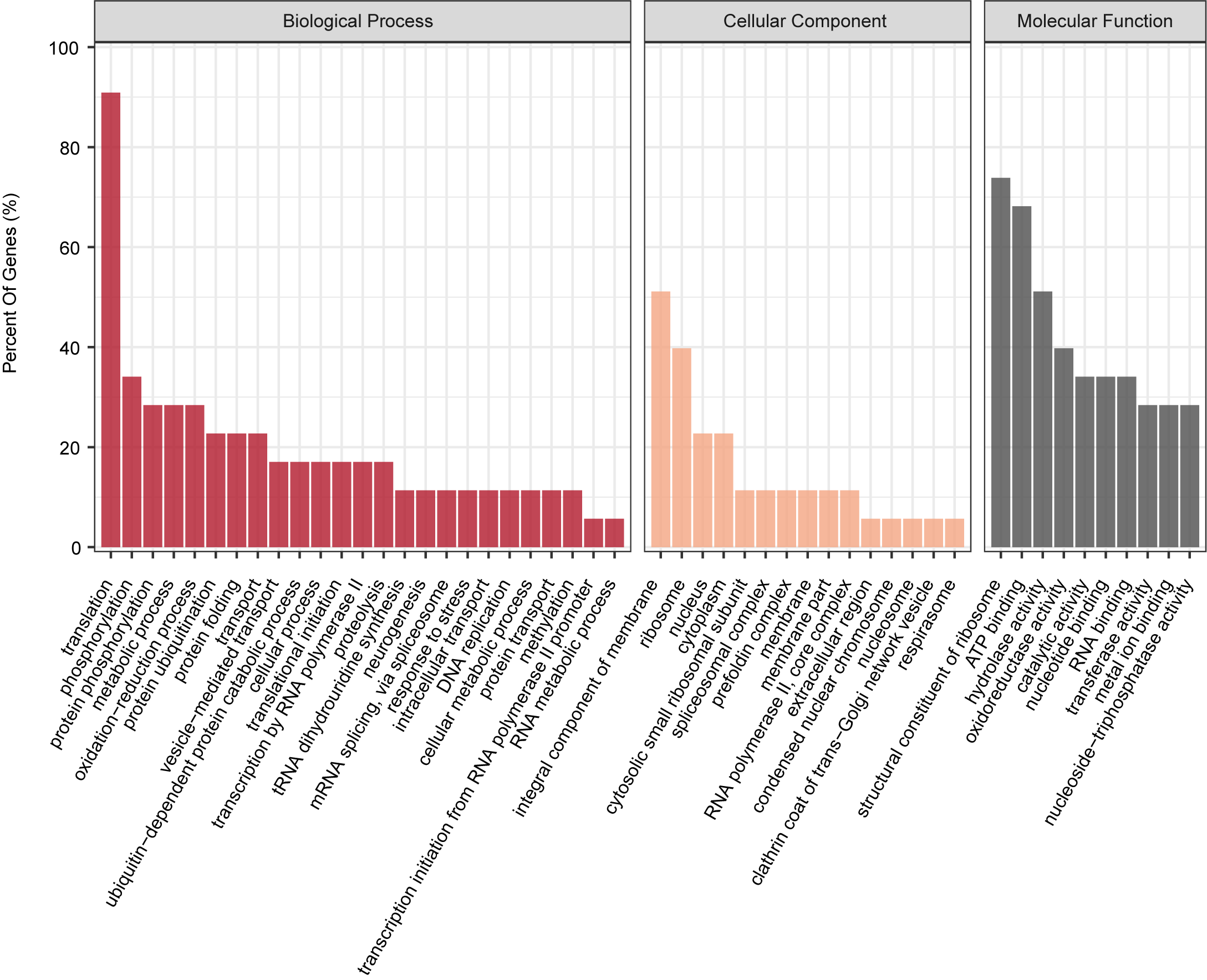

Supplement: Supplementary file 8 [file Image_1.TIF]

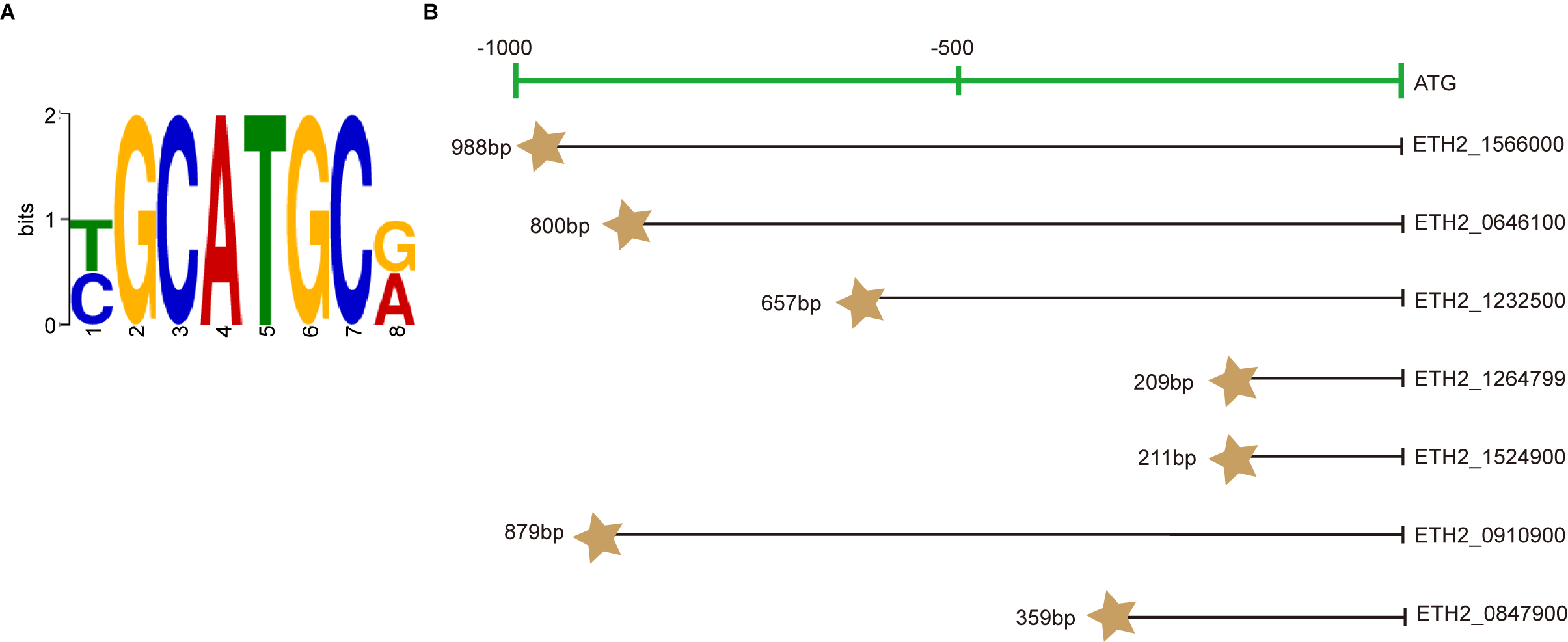

Supplement: Supplementary file 9 [file Image_2.TIF]
